# Treatment of Impetigo with Antiseptics—Replacing Antibiotics (TIARA) trial: a single blind randomised controlled trial in school health clinics within socioeconomically disadvantaged communities in New Zealand

**DOI:** 10.1186/s13063-022-06042-0

**Published:** 2022-02-02

**Authors:** Sarah Primhak, Alicia Gataua, Diana Purvis, John M. D. Thompson, Cameron Walker, Emma Best, Alison Leversha

**Affiliations:** 1 Paediatric Infectious Diseases, Starship Children’s Health, Auckland, New Zealand; 2 University of Auckland, Auckland, New Zealand; 3 National Hauora Coalition, Auckland, New Zealand; 4 Paediatric Dermatology, Starship Children’s Health, Auckland, New Zealand; 5 Community Paediatrics, Starship Children’s Health, Auckland, New Zealand

## Abstract

**Background:**

Impetigo is a common and contagious bacterial skin infection, affecting children worldwide, but it is particularly prevalent in socioeconomically disadvantaged communities. In New Zealand, widespread prescribing of the topical antibiotic fusidic acid had led to an increase in antimicrobial resistance of *Staphylococcus aureus*. Alternative treatments are urgently being sought, and as impetigo is a superficial infection, it has been suggested that topical antiseptics such as hydrogen peroxide or simple wound care alone may treat impetigo while avoiding the risk of increased antimicrobial resistance.

**Methods:**

This protocol for a non-inferiority, single-blind randomised controlled trial compares topical fusidic acid with topical hydrogen peroxide and with simple wound care in the treatment of childhood impetigo. Participants are randomised to one of the three treatments for 5 days. The primary outcome is clinical improvement assessed through paired photographs analysed by graders blinded to treatment arm. The trial is based in school health clinics in an urban centre in New Zealand. Comparison of antimicrobial resistance patterns pre- and post-treatment is also performed.

**Discussion:**

Special note is made of the need to involve the communities most affected by impetigo in the design and implementation of the clinical trial to recruit the children most in need of safe and effective treatments.

**Trial registration:**

Australian New Zealand Clinical Trials Registry (ANZCTR) 12616000356460. Registered on March 10, 2016

Protocol amendment number: 05

EB and AL contributed equally as senior authors.

**Supplementary Information:**

The online version contains supplementary material available at 10.1186/s13063-022-06042-0.

## Introduction

Impetigo is a contagious superficial bacterial skin infection, predominantly affecting children [1, 2]. It is described as one of the top 50 most common diseases [3], with a global prevalence estimated at 162 million [4]. New Zealand (NZ) is a high-income country in the South Pacific with a diverse population including tangata whenua- NZ indigenous population (Māori) who make up 17% of the population. During western colonisation, NZ established the Treaty of Waitangi [5] between the Crown and Māori which ensures a legal requirement to good governance and the right to equitable health outcomes for indigenous people [6]. While the Pacific peoples of NZ (those from the Polynesian and Melanesian Pacific islands surrounding NZ) are not part of the treaty agreement, they make up a significant proportion of NZ’s population (~ 7%), and there is recognition of significant inequities for both Māori and Pacific populations and the obligation to address these. Primary care is generally funded for children under 13 years of age, but socioeconomic barriers to care are still recognised. NZ experiences a particularly high burden of skin and soft tissue infections with as many as 11% of NZ children aged under 15 years consulting their primary care provider for skin infections annually. There is an inequity in the burden of disease with the highest rates seen in children of Māori and Pacific peoples.

As a superficial infection, impetigo is frequently treated topically rather than systemically. Since the early 2000s, New Zealand has experienced high prescribing rates of the topical antibiotic fusidic acid; rates of dispensing are highest for preschool children, followed by those age 75+ and 5–14 years. Dispensing is also highest in Pacific Island and Māori ethnicities [7, 8]. Following the increase in prescribing, a subsequent rise in fusidic acid resistant *S. aureus* isolates has been reported; resistance is now demonstrated in 28% of NZ *S. aureus* isolates [9]. In 2005, a fusidic acid resistant and methicillin resistant *S. aureus* (MRSA) clone was identified and has become the dominant MRSA clone in New Zealand [10]. This suggests that widespread community use of fusidic acid has not only led to selection of fusidic acid resistant clones but also concurrent MRSA. In an attempt to limit the development of further antimicrobial resistance by avoidance of topical antibiotics, NZ has updated national impetigo guidance. While oral antibiotics remain the first line for severe or multi-lesional impetigo, topical fusidic acid is no longer first line in mild-to-moderate disease, superseded by the advice to use an antiseptic cream, 1% hydrogen peroxide [11]. The UK National Institute of Clinical Excellence (NICE) have also released a draft guideline on impetigo proposing the same change [12]. These prescribing guidelines are based on evidence from a single randomised controlled trial performed in 1993 with no information provided on fusidic acid resistance rates within the study population. This demonstrated “a tendency towards somewhat lower efficacy in [hydrogen peroxide] compared to [fusidic acid]” with no significant difference between the groups [13]. Since that time, antimicrobial resistance patterns to fusidic acid have changed within NZ [7, 8]. and potentially within other countries where it is used topically or systemically.

In the past, clinical trials of impetigo have presented challenges and this trial protocol seeks to address these. Impetigo is predominantly a disease seen in the community with primary care presentations representing only a small proportion of cases. Many families never seek medical review, particularly in populations where financial concerns limit health-seeking behaviours [14]. A clinical impetigo trial should, therefore, ideally be based within a community setting. Impetigo is a disease of childhood, a challenging group to enrol in clinical trials with previous trials on impetigo treatment including large numbers of adults; the RCT upon which the current practice is based recruited patients with a mean age of 17 years [13].

Defining primary outcomes in impetigo treatment is also challenging as demonstrated by the variety of outcomes measures across prior clinical trials. A systematic review concluded that more robust outcome measures were required [15]. Time to reported clinical endpoints have ranged between 1 and 3 weeks after starting treatment. Some trials have used complete cure while others are satisfied with clinical improvement of varying definitions. Many are based on observer defined definitions, but even the more objective scores such as the Skin Infection Rating Score (SIRS) have been used as a primary outcome measure in a variety of ways, including an absolute reduction in score, predefined absolute decrease in score, or as a percentage decrease from the baseline [6, 16].

To best inform our practice, our trial requires a majority recruitment of children of Māori and Pacific children, the groups most affected by impetigo in NZ. There are marked inequities in health outcomes for Māori and Pacific Island children, and it is critical that health research is focused on understanding and addressing these inequities. Impetigo is contributing to inequity in health outcomes and therefore is crucially important to Māori and Pacific health. With the burden of skin disease affecting predominately Māori and Pacific children, appropriate cultural engagement with these communities was recognised as a key factor, both for successful trial completion and ensuring meaningful results which will be acceptable, generalisable, and implementable for those most affected by impetigo.

### Objectives

We hypothesize both simple wound care and topical hydrogen peroxide are non-inferior to topical fusidic acid in the treatment of impetigo.

Therefore, the aims of this clinical trial are:

1. To compare the effectiveness of topical fusidic acid with topical hydrogen peroxide and simple wound care in the treatment of mild-to-moderate impetigo in a community with both high rates of impetigo and increasing fusidic acid resistance.

2) To examine potential changes in the antimicrobial resistance of skin pathogens in response to these different treatments for impetigo.

### Trial design

TIARA is an open label, single-blind, non-inferiority randomised controlled trial with three parallel treatment groups. The primary endpoint is clinical improvement at seven days. Randomisation is performed 1:1:1 within each school clinic.

## Methods: participants, interventions, and outcomes

### Study setting

Auckland city has a temperate climate and is a large urban centre of 1.6 million people. Primary school health clinics serve the more socioeconomically disadvantaged areas within two of the three district health boards in the Auckland region: Auckland and Counties Manukau. These health clinics provide primary care, including free skin and throat infection management, to students aged 5–13 years [17, 18]. The school nurses running the clinics are invited to participate in this study to provide a potential eligible population of ~ 10,000 enrolled students. Due to their over representation of socio-economic disadvantage, over 90% of children in the schools are of Māori or Pacific Island ethnicity, and there is a high rate of impetigo.

### Eligibility criteria

Children meeting the eligibility criteria are identified and their caregivers contacted to explain the study and obtain consent. Caregivers must provide verbal informed consent before any study procedures occur. This is then followed by written informed consent (see Appendix 1 for sample of informed consent form).

#### Inclusion criteria

Children eligible for the trial must comply with all of the following prior to randomisation:

1. Enrolled in one of the participating school clinics
2. Mild-to-moderate impetigo
3. Aged 5–13 years

#### Exclusion criteria

1. Severe impetigo requiring oral antibiotics; defined as extensive lesions (> 3 lesions or > 5% body surface area), presence of cellulitis, or fever > 38.5 °C
2. Children who are immunocompromised
3. Known allergy to study drugs
4. Current use, or use within the previous 5 days, of topical or oral antimicrobials
5. Commencement of antimicrobials for other reasons during the trial period
6. Failure to obtain informed consent for randomisation or withdrawal of consent

Excluded children continue with treatment according to the existing school health clinic standard operating procedures (SOP).

### Intervention

All lesions are cleaned with saline and scabs gently removed. For the group randomised to fusidic acid, 2% fusidic acid ointment (DP Fusidic Acid, Douglas Pharmaceuticals Ltd, Auckland, NZ) is applied topically, and for the hydrogen peroxide arm, 1% hydrogen peroxide cream (Crystaderm, AFT Pharmaceuticals, Auckland, NZ) is applied topically. In both cases, an adequate amount to cover each lesion is used and dressing(s) then applied. A tube of appropriate topical medication is supplied for the child and/or caregivers to continue applications twice daily for 5 days with dressing changes. Participants allocated to simple hygiene measures receive no medication but a dressing is applied following the cleaning of the lesion(s). All participants are provided with supplies to allow them to clean and redress the wound twice daily for 5 days. Low adherent wound pads are used so as not to interfere with the wound healing process. In all groups, scabies is treated if present.

### Modifications

All adverse effects, including pain, itch, or allergy to study medication, will be reported; the study medication must be withdrawn and the patient changed to routine treatment as per the SOP of the health clinic. If clinical deterioration while on study medication is identified by the school nurse, the study medication may be withdrawn at the nurse’s discretion. In this case, the patient will be changed to routine treatment as per the standard operating procedures of the health clinic.

### Adherence

Face-to-face reminders of adherence are provided by nurses at both day 0 and day 2 visits, and adherence over the trial period is assessed on days 2 and 7. Sticker charts are provided for each participant to encourage adherence.

### Participant timeline

**Fig. 1:**
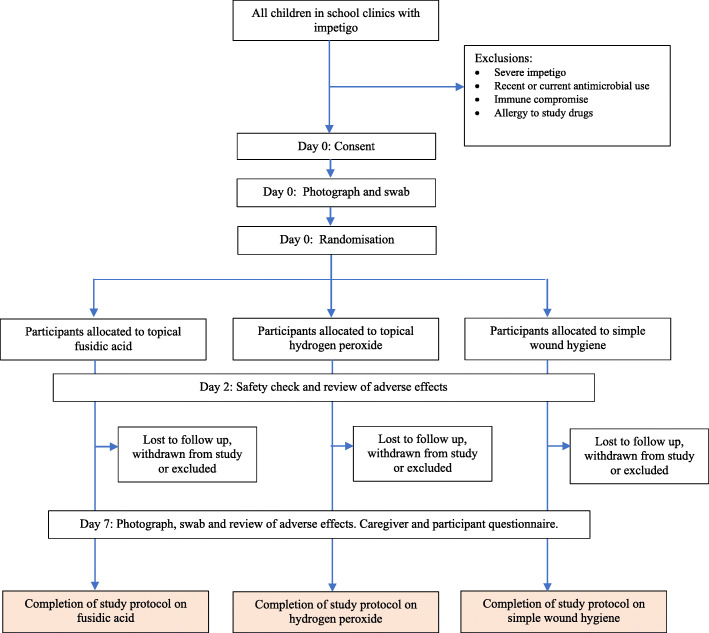
Schedule of enrolment, interventions, and assessments

**Fig. 2:**
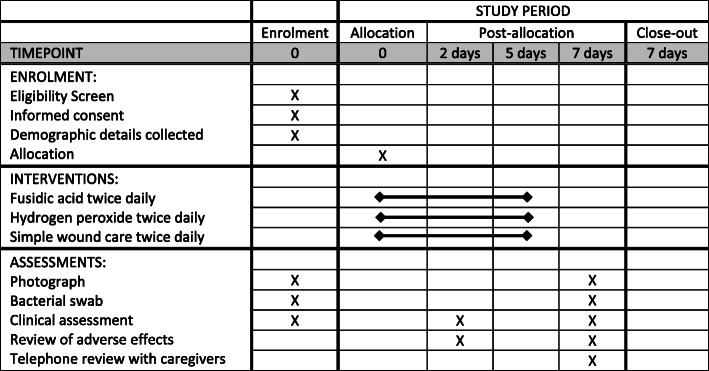
SPIRIT diagram

On the first visit (day 0), demographic data, inclusion and exclusion criteria, and verbal consent are obtained. All lesions are cleaned and the single largest lesion is photographed using a digital camera, and a bacteriological swab is taken from the same lesion as the photograph. The patient is then randomised and the appropriate treatment is commenced and continued for 5 days. Two days after enrolment into the trial (day 2), a safety check is performed by the school nurse to assess for rapid worsening of the impetigo or for adverse effects. The safety check can be performed between days 2 and 4 if necessary to allow for day 2 falling on a weekend. Caregivers are asked to contact the school nurse between visits, if they have concerns about the lesion getting worse. Seven days after commencing the trial (day 7), the participant is re-assessed by the nurse. A second set of photographs and repeat bacterial swab is taken from the same lesion as originally documented. Both child and caregiver complete a verbal questionnaire. Caregivers can withdraw a participant from the study at any stage. For the full schedule of interventions and assessments see Figs. 1 and 2.

### Sample size

On the assumption of non-inferiority between hygiene measures and topical fusidic acid and between topical hydrogen peroxide and fusidic acid, and a predicted efficacy of fusidic acid of 80%, we require 160 patients in each intervention group. This provides 80% power and a one-sided *α* of 0·05 to show non-inferiority (10% margin) between each group and topical fusidic acid. In order to allow for 10% loss to follow-up and subsequent exclusions, recruitment of 178 participants to each group is required. Subsequent exclusions and loss to follow-up are defined as participants not available for follow-up at day 7 or when two digital images are not available to assess. Estimates of efficacy are extrapolated from published data [6, 19, 20], and the 10% margin was considered a clinically significant difference [21].

### Recruitment

School health teams within recruiting schools will follow existing SOPs. Community health workers attend every class 3–5 days each week during the school term and ask children to self-identify any skin infections. These children are then reviewed by the school nurse. If the lesions are confirmed as impetigo, then eligibility for participation is assessed.

### Allocation concealment mechanisms and implementation

Randomisation is implemented by block randomisation within each participating school, sequence allocation code was written in R [22]. Children are randomly allocated (1:1:1) to topical fusidic acid, topical hydrogen peroxide, or simple hygiene measures. Participating schools are randomised separately to limit bias caused by excess recruitment to any individual arm within a single school*.* Allocation is performed by school nurses using the pre-generated codes contained in sealed, opaque, sequentially numbered envelopes. Both the participant and school nurse are aware of treatment allocation due to the appearance of the study medications (e.g. hydrogen peroxide has a silvery sheen) or lack of study medication in the simple hygiene measure arm. However, investigators, photograph reviewers, and laboratory staff are blinded to allocation.

## Data collection methods

### Demographic characteristics

Baseline characteristics of participants are collected at the time of randomisation and reported per randomisation group. The following demographic characteristics will be reported: age, gender, ethnicity, weight, history of pre-existing skin disease, allergies, and location of most severe lesion. All data is collected directly onto an electronic database used by school health clinics; additional trial questions have been created and added to this database by the study investigators. An equivalent paper proforma was designed by SP and included in the study materials for use if required.

### Primary outcome methods

The primary outcome of this trial is treatment success based on comparison of digital photographs taken at days 0 and 7. If clinical assessment leads to early discontinuation of the trial medication, this is also considered unsuccessful treatment. To aid standardisation, the primary outcome uses digital photographs independently assessed by blinded reviewers. The single largest lesion is photographed prior to commencing treatment, using a digital camera at a distance of 15 cm with an adhesive paper tape measure and unique study ID placed next to the lesion. Three digital images are taken at each data collection visit, and all are submitted to the study investigators. The single best quality image is then selected by the lead investigator (SP) for outcome assessment. Pairs of images are presented to three individual assessors blinded to intervention arm. The method of image presentation is randomised such that assessors are unaware of which image was taken first and which second. The outcome will be treatment success if the images are considered healed or improved and treatment failure if they are the same or worse, or if the reviewers cannot determine the outcome based on the digital images (e.g. due to poor quality images). Where there is discordance between assessor opinions, the majority opinion of two out of three reviewers will be considered the correct assessment. Patients removed from the trial protocol by school nurses or general practitioners before 7 days due to clinical deterioration will also be considered treatment failures. This process has been standardised previously in another large RCT of treatment of impetigo in remote access locations; different from our multicentre urban setting [21].

### Clinical assessments

Clinical assessments are standardised and recorded on the existing skin assessment database used for school health clinics. A written description of the location of the lesions and identification of the primary lesion being used for assessment is recorded. A paper template is also provided to allow nurses the option to mark on a diagram the location of the lesions.

Seven days after commencing the trial (day 7), the participant is re-assessed by the school nurse, who also records whether the lesion has improved. Both child and caregiver are asked to comment on their satisfaction with treatment and any adverse events related to the medication, including itch, pain, or allergy. School records are checked for absence over the prior 7 days.

### Microbiology

All children have a bacteriological dry cotton swab taken from the most severe lesion at presentation and at day 7. Swabs are cultured onto blood agar and any clinically significant growth is reported. The European Committee on Antimicrobial Susceptibility Testing (EUCAST) susceptibility method and criteria are used for *S. aureus* susceptibility testing. Susceptibility is tested to commonly used skin and soft tissue antimicrobials; fusidic acid, flucloxacillin, erythromycin, clindamycin, and co-trimoxazole. If MRSA is identified then extended susceptibility is performed, including mupirocin and tetracyclines. No susceptibility testing is performed on *S. pyogenes*.

## Outcomes

### Primary outcome

The primary outcome of this trial is treatment success assessed by comparison of digital photographs or clinical deterioration based either on digital images or on nursing assessment leading to discontinuing trial medication.

### Secondary outcomes

- Clinical success will be compared between groups. This will be defined by
  - Nursing opinion that the impetigo has improved at day 7
  - Participant and/or caregiver opinion that the impetigo has improved at day 7
- Microbiological secondary outcomes are eradication of *S. pyogenes* and/or *S. aureus* on day 7 and development of antibiotic resistance on day 7 compared to baseline
- Educational impact is assessed by comparison of school absence over the 7 days of the trial period
- Adverse events will be compared across study arms

### Retention

A supermarket voucher worth NZ$20 ise offered to the family as *koha*, a thank you gift on successful completion of the trial protocol.

### Data management

Trial data is stored using a study identification number on a password protected access database maintained on a secure network. This database is also used to randomise images and presents anonymised pairs of photographs to the graders for analysis, and records the outcomes of the grading.

### Statistical analyses

For baseline data, d**i**chotomous variables will be summarised as proportions of patients in each treatment group, differences between groups will be assessed using a chi-square statistic, and where small cell sizes (less than 5) are present, a Fisher’s exact test will be used. Continuous variables with an underlying normal distribution will be summarised as mean and standard deviation, and differences between groups will be assessed using Student’s *t*-test. Other distributions will be either transformed if suitable and *t*-tests performed with reporting of geometric means or distributions will be reported as median and interquartile range and differences in groups assessed using Wilcoxon rank non-parametric tests with Hodges-Lehmann estimates and 95% confidence intervals.

Analysis will be performed after completion of recruitment. Both hydrogen peroxide and simple hygiene groups will be compared independently with fusidic acid with 95% confidence interval. Non-inferiority will be defined as a treatment success rate of no more than 10% below that of the fusidic acid success rate. An intention to treat and per protocol analysis will be performed using all patients with available primary outcome data. Patients without primary outcome data or for whom caregivers withdrew consent to participate will not be included in the analysis.

To investigate predictors of treatment success, backwards stepwise random-effects logistic regression will be performed on a priori and other variables identified as different in baseline characteristics between randomisation groups.

### Data monitoring

A data safety monitoring board (DSMB) has been convened. This comprises an international expert in impetigo, a local expert in paediatric infectious diseases, and a statistician. An interim safety analysis blinded to allocation will be performed after recruitment of 150 participants. If concerns are expressed, the unblinded data may be made available to the DSMB on request. Stopping criteria may include slow accrual, poor data quality, unacceptable adverse events, and emerging information that makes the trial irrelevant. The DSMB will discuss the outcome of the analysis with the trial steering committee. The unblinded data, apart from the outcome of the DSMB decision, will not be made available to the authors prior to the completion of the trial and unblinding.

### Harms

Any adverse event will be reported; these are defined as any untoward medical occurrence in a subject without regard to the possibility of a causal relationship after entry into the study and until the completion of the study. At day 2 and day 7 of the trial, a safety check is performed by the school nurse to assess for rapid worsening of the impetigo or for adverse effects. If these occur, then the participant can be withdrawn from the trial at the discretion of the nurse. On day 2 and day 7, participants and/or caregivers are directly questioned regarding specific harms including itch, pain, redness, or any other adverse effects of medication. Any requirement for additional medical intervention is considered a potential harm. All harms will be reported.

### Auditing

Regular visual review of the data will be performed by the lead investigator (SP) for completeness and quality of the data.

## Discussion

The aim of this study is to investigate the relative effectiveness of non-antibiotic management of impetigo in children. The use of existing school health clinics located within the more socioeconomically deprived communities in Auckland, NZ, allows access to a large population of children within a community who might not otherwise seek medical help. This ensures access to an appropriate cohort of children at high risk of impetigo.

Engaging authentically with Māori and Pacific communities is vital  to the success of this project. As outlined above, Māori and Pacific communities living in New Zealand face barriers to accessing appropriate and timely primary healthcare for potentially preventable conditions such as impetigo [14]. Research is critical to address these inequities and should be undertaken in a culturally appropriate way using a partnership approach with Māori and Pacific researchers. School nurses who are known and trusted by the families, and representative of the school communities, will be important to ensure effective engagement.

The National Hauora Coalition (NHC), a Māori primary health organisation, was consulted early in study design. A Māori nurse leader (AG) directed key trial implementation and engendered the support of the school nurses who represent both Māori and Pacific Island healthcare workers. Information leaflets have been provided in multiple languages, including Te Reo Māori, Tongan, and Samoan, and nursing staff provide additional verbal information as a more acceptable communication [14]. Specific acts of recognition and practical reimbursements have been shown to contribute significantly to a sense of value and reduce the financial strain when accessing healthcare [14]. To acknowledge this, a gift (koha) of a NZ$20 supermarket voucher is offered to each family after completion of the trial.

Because of the large and disparate pool of recruiters, it is important that the primary outcome is as comparable and unbiased as possible. Digital images allow for centralised assessment despite the distance between recruiting sites. However, this means the existing scoring system (SIRS) is not practical as variables included in the scoring such as warmth, pain, and itch are not amenable to visual assessment alone. For this reason, assessor defined scoring was used. Erythema is also one of the elements of the SIRS scoring system, and when assessing children with darker skin, this is often underestimated [23], leading to minimisation of the severity of lesions in those with darker skin. The use of digital images and multiple reviewers for the primary outcome aims to minimise the bias inherent in the majority of previous studies on impetigo and provide a reproducible outcome, consistent with real world improvement. This remains an imperfect measure as it is dependent on good quality digital images and reviewer defined assessment. Due to the visible difference in the topical medications, neither school nurses nor participants could be blinded to treatment. This introduces a potential element of bias for those removed from the study early due to clinical treatment failure. It was considered unfeasible to introduce a placebo medication to the simple wound hygiene group. Potentially, any ointment without antimicrobial properties could provide an environment for bacterial growth or negatively affect bacterial growth, potentially changing the outcome for this group.

Topical antibiotics remain the mainstay of treatment for mild-to-moderate impetigo in many countries around the world, including New Zealand. Widespread community use and a tendency for prolonged courses mean that they excel at inducing antimicrobial resistance. Evidence for antiseptic use or simple hygiene measures in impetigo is extremely limited: this will be only the second trial to compare these topical antiseptics and antibiotics and the first to take current antimicrobial resistance patterns into account. On the background of a worldwide increase in antimicrobial resistance and with increasing recognition of the importance of antimicrobial stewardship, it is timely to re-address the evidence for the role of antiseptics and antibiotics in the treatment of impetigo. This is only possible, however, if the most affected communities are appropriately engaged in this research.

## Supplementary Information

**Additional file 1: Supplemental Table 1.** Trial registration data.

**Additional file 2.** Appendix 1 Consent form.

## Data Availability

Patient consent/assent form is attached. Materials including patient information and data collection sheets are available on request from the authors.
