# Supplementary material for: Treatment of Impetigo with Antiseptics—Replacing Antibiotics (TIARA) trial: a single blind randomised controlled trial in school health clinics within socioeconomically disadvantaged communities in New Zealand
Source: Trials. 2022 Feb 2;23:108. doi: 10.1186/s13063-022-06042-0 (PMC8812233; doi:10.1186/s13063-022-06042-0)
Supplement: Supplementary file 1 — Additional file 1: Supplemental Table 1. Trial registration data. [file 13063_2022_6042_MOESM1_ESM.docx]

**Supplemental Table 1. Trial registration data**

| Data category | Information |
| --- | --- |
| Primary registry and trial identification number | Australian New Zealand Clinical Trials Registry (ANZCTR) 12616000356460 |
| Date of registration in primary registry | 18/03/2016 |
| Sources of monetary or material support | Cure Kids New Zealand, Auckland District Health Board charitable trust (A+), Tupu Research Grant, Ko Awatea, The New Zealand Society Wound Care Grant, The Joan Mary Reynolds Charitable Trust, The Starship Foundation and The National Health Research Council of New Zealand |
| Primary sponsor | University of Auckland |
| Contact for public enquiries | SP, spri856@aucklanduni.ac.nz, EB, AL |
| Contact for scientific enquiries | SP, EB, AL, University of Auckland |
| Public title | Treatment of Impetigo with Antiseptics- Replacing Antibiotics (TIARA) Trial |
| Scientific title | Treatment of Impetigo with Antiseptics- Replacing Antibiotics (TIARA) Trial- a single blind randomised controlled trial in school health clinics within socioeconomically disadvantaged communities in New Zealand |
| Countries of recruitment | New Zealand |
| Health condition or problem studied | Topical antibiotic, antiseptic, impetigo |
| Interventions | Standard of care: Fusidic acid antibiotic (fusidic acid cream twice daily) |
|  | Comparator 1: Hydrogen peroxide antiseptic (hydrogen peroxide cream twice daily) |
|  | Comparator 2: Simple wound care with dressing |
| Key inclusion and exclusion criteria | Ages eligible for study: 5 years to 13 years 364 days, genders eligible: al |
|  | Inclusion criteria: mild to moderate impetigo, attending school health clinic |
|  | Exclusion criteria: Severe impetigo requiring oral antibiotic, immunocompromise, allergy to study drug, current or recent us of antimicrobials, commencement of antimicrobials for other reasons during the trial period, failure to obtain informed consent for randomisation or withdrawal of consent |
| Study type | Interventional |
|  | Allocation: randomised; Intervention model: parallel, 3 arms, Masking: single blind, Analysis: Non inferiority |
|  | Primary purpose: treatment |
| Date of first enrolment | June 2017 |
| Target sample size | 480 |
| Recruitment status | Recruiting |
| Primary outcome | Improvement of impetigo based on photographs at 7 days |
| Key secondary outcomes | Clinical success defined by nursing opinion and participant and/or caregiver opinion; Eradication of *S. pyogenes* and/or *S. aureus* on day 7 and development of antibiotic resistance on day seven compared to baseline; School absence over the seven days of the trial period |
