# Supplementary material for: Treatment of Impetigo with Antiseptics—Replacing Antibiotics (TIARA) trial: a single blind randomised controlled trial in school health clinics within socioeconomically disadvantaged communities in New Zealand
Source: Trials. 2022 Feb 2;23:108. doi: 10.1186/s13063-022-06042-0 (PMC8812233; doi:10.1186/s13063-022-06042-0)
Supplement: Supplementary file 2 — Additional file 2. Appendix 1 Consent form. [file 13063_2022_6042_MOESM2_ESM.pdf]

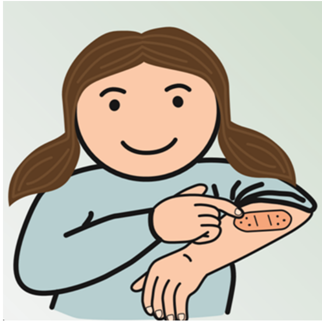

Finding the best way to treat school sores

Whānau/family Information Sheet

Locality:	**Auckland and Manakidz**	Ethics committee ref.: **16/NTA/113**
Lead investigator:	**Dr. Alison Leversha**	Contact phone number: **+64 21 629 047**

You and your whānau are invited to take part in a study to find out the best way to treat school sores (also known as Impetigo). Whether or not you take part is your choice. If you don't want your child to take part, you don't have to give a reason, and it won't affect the care your child receives. If you do want to take part now, but change your mind later, you can pull out of the study at any time.

This Information Sheet will help you decide if you'd like to take part. It sets out why we are doing the study, what is involved, what the benefits and risks to you might be, and what would happen after the study ends. We will go through this information with you and answer any questions you may have. You do not have to decide today whether or not you will take part in this study. You may want to talk about the study with other people, such as family/whānau, friends, or healthcare providers before you decide. Feel free to do this.

If you agree to take part in this study, you will be asked to sign the Consent Form at the end of this document. You will be given a copy of both the Information Sheet and the Consent Form to keep. This document is 5 pages long, including the Consent Form. Please make sure you have read and understood all the pages.

WHAT IS THE PURPOSE OF THE STUDY?

Kia ora and Pacific greetings. I am the school nurse that works in the health clinic at your school. We have many school clinics across Auckland run by Manakidz and Starship. We provide free sore throat checks, treat skin infections and help prevent rheumatic fever. You signed a form already saying you agree to us providing this service for your child. We would like to invite you and your whānau/family to help us do something else.

School sores are common and there are many ways of treating them. Before we started our clinics in your school, most school sores were left to get better on their own. Some did get better, but some got much worse and ended up being so bad children had to go to hospital. School sores can also be treated by antibiotic tablets or syrup. This is best for large skin infections as they kill the germs and stop nasty complications. However, for small and medium sized school sores, we don't know exactly what to use: really good cleaning and dressings, antiseptic cream or antibiotic cream. The antibiotic cream is free when you get it from us, but there is worry it may be causing resistance (i.e. the germs will no longer be killed by them and we will therefore have to use more powerful antibiotics). The antiseptic cream costs a lot from the pharmacy and the government won't pay for it until we can show it works. And for small infections, maybe we just need to have really good cleaning and care. This study is all about working out the best way to treat school sores so we get the best results with the least problems.

WHAT WILL MY PARTICIPATION IN THE STUDY INVOLVE?

Your child is already able to receive free checks and treatment at the health clinic at their school. Today, when we asked if anyone had a sore, your child put their hand up. The nurse checked your child in the clinic and saw that they have small to medium sized school sores that would be suitable to be part of this study finding out the best way to treat school sores.

If you agree for your child to take part in this study, we will take a photo of the sore, take a swab to see what germs are causing it, and then randomise your child to receive one of three different treatments. Randomise means we don't choose which treatment: it is written down in a folder and which treatment your child gets is by chance, like flipping a coin.

Your child will get one of 3 treatments:

1. Really good cleaning, covering with a dressing
2. Cleaning, covering with antiseptic cream (Crystacide), then covering with a dressing

or

3. Cleaning, covering with antibiotic cream (Foban), then covering with a dressing

To help the school sores get better, the treatment needs to be done twice a day for 5 days. We will show you how to do it and will provide you with all the medicine and plasters you need. We will check the sore is getting better in a couple of days and then again in a week. We will also take another photo and another swab at the end to check how well the treatment has gone.

WHAT ARE THE POSSIBLE BENEFITS AND RISKS OF THIS STUDY?

Your child will continue to get free checks and treatment whether or not they are in the study. Whilst we believe there are going to be no risks to being in this study, there is a small chance the treatment option that your child gets randomised to won't work as well as we hoped. At any stage, if the sore gets worse, we will go back to using more powerful treatment. There is also a small chance the antiseptic cream might cause some stinging which is why we will be checking with you and your child how things are going.

WHO PAYS FOR THE STUDY?

There are no costs for being in this study. We pay for all the medicine and dressings. There is also no cash payment for being in the study. The study is funded by Cure Kids and involves us from Starship Hospital, Manakidz and the local schools all working together. If you have any questions about the project or wish to know more, please contact the lead investigator Dr. Alison Leversha. The contact details are at the end of this information sheet.

WHAT IF SOMETHING GOES WRONG? WHAT ARE MY RIGHTS?

If you decide that you do not want to be involved with the study, or want to withdraw at any stage that is your right. You may withdraw without any disadvantage. You can tell the nurse or community health worker or whanau support worker of your decision to withdraw.

If you take part, you have the right to ask for a copy of the photos. The results from the research will be sent back to you and your whānau through summary sheets (if you want them) and community presentations/hui.

No material that could personally identify you (let other people know who you are) will be used in any reports on this study. The identity of any person involved will be kept confidential. Information collected from the study will be confidentially kept for 10 years after your child turns 16 years. After this time, all data, pictures and samples will be destroyed.

In the unlikely event of a physical injury as a result of participation in this study, you may be covered by ACC. ACC cover is not automatic and the case will need to be assessed by ACC according to the provisions of the 2002 Injury Prevention Rehabilitation and Compensation Act. There is no cover for mental injury unless it is a result of physical injury.

WHAT HAPPENS AFTER THE STUDY OR IF I CHANGE MY MIND?

The study will finish in December 2018. Information from the study will be used to help doctors and nurse to know what treatment to use at different stages of skin sores. It will also be used to help the government decide if we should keep using Foban or if we should change to using antiseptic cream. If this was the case, you could get Crystacide free instead of having to buy it from the pharmacy.

WHO DO I CONTACT FOR MORE INFORMATION OR IF I HAVE CONCERNS?

If you have any questions, concerns or complaints about the study at any stage, you can contact:

Dr. Alison Leversha, Community Paediatrician, Starship Children's Hospital
Email: alisonl@adhb.govt.nz
Telephone **+64 21 629 047**

Ms XXe, Project Nurse, Starship Children's Hospital
Email: ....@adhb.govt.nz
Telephone: **xxxxxxxxx**

Ms XXe, Project Nurse, Manakidz
Email: ....@middlemore.co.nz
Telephone: **xxxxxxxxx**

If you have any questions or complaints about the study you may contact the Auckland and Waitematā District Health Boards Maori Research Committee or Maori Research Advisor by telephoning 09 4868920 ext 3204.

If you require Māori cultural support talk to your whānau in the first instance. Alternatively, you may contact the administrator for He Kamaka Waiora (Māori Health Team) by telephoning 09 486 8324 ext 2324.

If you have any questions or concerns about your rights from taking part in this research study you can contact an independent health and disability advocate. This is a free service provided under the Health and Disability Commissioner Act.
Telephone: (NZ wide): 0800 555 050
Free Fax (NZ wide): 0800 2787 7678 (0800 2 SUPPORT)
Email (NZ wide): advocacy@hdc.org.nz

If you have any medical questions or health concerns, please contact Healthline (a free service with trained nurses to give you advice): 0800 611 116

The study will commence in October 2016 and finish in December 2018. The study was approved by the Health and Disability Ethics Committee on xx/x/2016 for 2 years, reference number **16/NTA/113**.

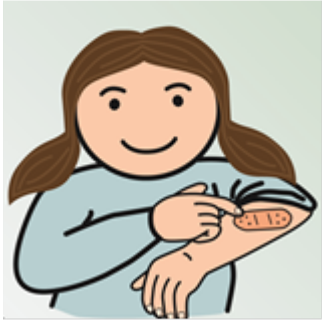

Finding the best way to treat school sores

Whānau/caregiver Consent Form

If you need an INTERPRETER, please tell us.

Please tick to show you consent to the following

I have read the Information Sheet, or have had it read to me in my first language, and I understand what it says.	Yes ☐
I have been given enough time to consider whether or not to participate in this study.	Yes ☐
I have had the opportunity to use a legal representative, whānau / family support or a friend to help me ask questions and understand the study.	Yes ☐
I am happy with the answers I have been given regarding the study and I have a copy of this consent form and information sheet.	Yes ☐
I understand that taking part in this study is voluntary (my choice) and that I may withdraw from the study at any time without this affecting my child's care.	Yes ☐
I consent to the research staff collecting and processing information about my child's health.	Yes ☐
I agree to a swab being taken from my child's sore now and in 7 days' time.	Yes ☐
I consent to the researchers storing the specimens from my child's skin swabs for up to 5 years, for later use as a part of this study.	Yes ☐
I agree to a photo being taken of my child's sore now and in 7 days' time.	Yes ☐
If I decide to withdraw my child from the study, I agree that the information collected about them up to the point when I withdraw may continue to be processed.	Yes ☐
I understand that taking part in this study is confidential and that no material, which could identify me, my child/children or my whānau/family personally, will be used in any reports on this study.	Yes ☐
I understand that the results will be shared with the family doctor.	Yes ☐
I know who to contact if I have any questions about the study in general.	Yes ☐

I wish to receive a summary of the results from the study (to be sent out to the mail or email address provided below)	Yes ☐	No ☐

My mail address is (only complete if you wish to receive a summary of the results of the study):

____

____

Declaration by parent/caregiver of child:

I consent to my child taking part in this study. Yes ☐ No ☐

Parent's name: ____

Signature: ______________________ Date: ______________________

Child's name: ____

Declaration by member of research team:

I have given a verbal explanation of the research project to the participant, and have answered the participant's questions about it.

I believe that the participant understands the study and has given informed consent to participate.

Researcher's name: ____

Signature: ______________________ Date: ______________________

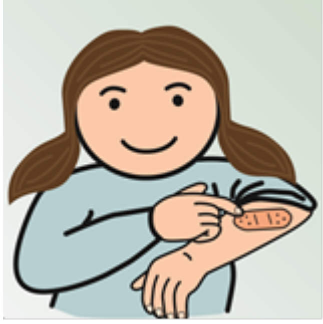

Finding the best way to treat school sores

Assent Form

The school nurse has talked to me and I know about the school sore study.

I agree to having photos taken of my sores.

I agree to having swabs taken of my sores.

I agree to having one of 3 different treatments for my sores.

Assent by child:

I am happy to take part in this study. Yes ☐

Child's name: ______________________________

Signature: ____________________ Date: ____________________

Declaration by member of research team:

Researcher's name: ______________________________

Signature: ____________________ Date: ____________________
